# Microbiological Contamination at Workplaces in a Combined Heat and Power (CHP) Station Processing Plant Biomass

**DOI:** 10.3390/ijerph14010099

**Published:** 2017-01-21

**Authors:** Justyna Szulc, Anna Otlewska, Małgorzata Okrasa, Katarzyna Majchrzycka, Michael Sulyok, Beata Gutarowska

**Affiliations:** 1 Institute of Fermentation Technology and Microbiology, Lodz University of Technology, Łódź 90-924, Poland; justyna.szulc@p.lodz.pl (J.S.); anna.otlewska@p.lodz.pl (A.O.); beata.gutarowska@p.lodz.pl (B.G.); 2 Department of Personal Protective Equipment, Central Institute for Labour Protection, National Research Institute, Łódź 90-133, Poland; kamaj@ciop.lodz.pl; 3 Center for Analytical Chemistry, Department of Agrobiotechnology (IFA-Tulln), University of Natural Resources and Life Sciences, Konrad Lorenz Str. 20, Tulln, Vienna (BOKU) 3430, Austria; michael.sulyok@boku.ac.at

**Keywords:** plant biomass, workplace bioaerosols, harmful biological agents, filtering facepiece respirators (FFRs), airborne dust

## Abstract

The aim of the study was to evaluate the microbial contamination at a plant biomass processing thermal power station (CHP). We found 2.42 × 10^3^ CFU/m^3^ of bacteria and 1.37 × 10^4^ CFU/m^3^ of fungi in the air; 2.30 × 10^7^ CFU/g of bacteria and 4.46 × 10^5^ CFU/g of fungi in the biomass; and 1.61 × 10^2^ CFU/cm^2^ bacteria and 2.39 × 10^1^ CFU/cm^2^ fungi in filtering facepiece respirators (FFRs). Using culture methods, we found 8 genera of mesophilic bacteria and 7 of fungi in the air; 10 genera each of bacteria and fungi in the biomass; and 2 and 5, respectively, on the FFRs. Metagenomic analysis (Illumina MiSeq) revealed the presence of 46 bacterial and 5 fungal genera on the FFRs, including potential pathogens *Candida tropicalis*, *Escherichia coli*, *Prevotella* sp., *Aspergillus* sp., *Penicillium* sp.). The ability of microorganisms to create a biofilm on the FFRs was confirmed using scanning electron microscopy (SEM). We also identified secondary metabolites in the biomass and FFRs, including fumigaclavines, quinocitrinines, sterigmatocistin, and 3-nitropropionic acid, which may be toxic to humans. Due to the presence of potential pathogens and mycotoxins, the level of microbiological contamination at workplaces in CHPs should be monitored.

## 1. Introduction

The advancement of civilization has prompted the identification of alternatives to fossil fuel, which have potentially unlimited reserves, yet are environmentally friendly. In accordance with the provisions of the United Nations Framework Convention on Climate Change and the 1998 Kyoto Protocol on the reduction of carbon dioxide, the use of renewable energy sources is being intensified globally [1]. The countries of the European Union and United States are among the global leaders in the production and consumption of renewable energy [2]. According to Directive 2009/28/EC of the European Parliament and of the Council, biogas and biomass are classified as renewable sources [3]. Between the years 2004 and 2014 global consumption of biomass in the heat, power, and transport sectors increased by 20% to an estimated 55.6 EJ [4]. Over the course of 2015, in the U.S. alone an estimated 1.4 × 10^3^ TWh of electricity was generated from biomass [5].

There are different technologies for biomass processing, depending on its intended use (e.g., heat or energy production) and type (mainly wood as well as wood, communal, agricultural, animal, and food processing waste) [6]. They include direct combustion, thermochemical (gasification, pyrolysis, supercritical fluid extraction and direct liquefaction), biochemical and agrochemical processes. It is estimated that processing of biomass for the purpose of energy production provides jobs for 822,000 people worldwide [7]. In all biomass-processing plants, workers are exposed to the harmful effects of organic dust [8]. In particular, the exposure concerns workplaces where grinding equipment and boilers are operated and where tasks of loading, unloading and transporting of plant biomass are carried out [9].

Organic dust released during the processing of plant biomass can be a source of harmful biological agents as it contains mineral substances, organic plant fragments, macro- and micro- organisms together with the substances secreted by them (bacterial endotoxins, glucans, mycotoxins and volatile organic compounds). After entering the respiratory system, these factors can cause irritation, and toxic, allergic, carcinogenic or fibrotic reactions resulting in diseases such as chronic obstructive lung disease, bronchial asthma, chronic bronchitis, bronchial hyperresponsiveness and organic dust toxic syndrome. Furthermore, they can cause mucous membranes irritation of the skin and the mucous membrane of the conjunctiva [10,11]. An epidemiological study showed that the exposure level to microorganisms has an impact on the occurrence of respiratory symptoms among biofuel workers [12]. Due to the high content of an easily assimilable carbon source, microorganisms can proliferate directly in plant biomass and can be transported with organic dust in the air. These are mainly mesophilic bacteria, actinomycetes and mould fungi, including pathogenic and toxinogenic species, such as *Aspergillus fumigatus* [13]. Fragmentary data on microbiological contamination at workplaces in plants processing plant biomass can be found in the literature [9,14]. However, no data exists on the potential sources of harmful biological agents or on the content of mycotoxins in workplaces where plant biomass is processed.

A solution for reducing the exposure of workers to the actions of harmful agents during biomass processing is the use of filtering facepiece respirators (FFRs). Nowadays, due to lower operating costs and reduced waste generation, reusable filtering half masks are becoming increasingly popular. During use, the exhalation of humid air and sweat increase the moisture content in the filter material. This together with the organic and inorganic substances deposited during air filtration, create ideal conditions for the growth of microorganisms [15,16,17]. Consequently, rather than protecting workers’ respiratory tracts, reusable filtering half masks may expose them to direct contact with biofilm and microbial toxins, including mycotoxins, present in the filtering material. At present, no studies on the subject of microorganisms colonising reusable filtering facepiece respirators (FFRs) used in power plants have been conducted. Due to the possibility of biofilm formation on the filtering material, its porous structure and the presence of unculturable microorganisms, such studies should be performed not only using culture methods, but also utilising microscopy and metagenomics.

The aim of this study was to evaluate the microbiological contamination in workplaces at a plant biomass processing power plant. Potential sources of harmful agents to employees’ health including bioaerosols, biomass and half masks utilized by the workers were taken into account. The scope of the research included the characterisation of the microclimate parameters and airborne dust in selected workplaces to determine the concentration and types of microorganisms in the air, in plant biomass processed in combined heat and power (CHP) stations and in the reusable FFRs. Microbial contamination of the reusable FFRs was verified using culture, microscopy and metagenomic methods. We also determined the content of secondary microbial metabolites in the processed plant biomass and in the reusable filtering half masks.

## 2. Materials and Methods

### 2.1. Workplaces in the Plant Biomass Processing Power Plant

The study was conducted in a 557 MW thermal power station, located in Poland. Four workplaces, at risk of exposure to harmful biological agents, were selected to study the microbial contamination and concentrations of organic dust in the air. The characteristics of these workplaces are summarised in Table 1.

The measurements were made in March 2015 (immediately prior to microbiological analyses) at a height of 1.5 m from ground level, in triplicates for each location.

Temperature, relative humidity and airflow rate were measured using thermo-anemometer VelociCalc^®^ Multi-Function Velocity Meter 9545 (TSI, Shoreview, MN, USA).

Airborne dust concentration was measured using a portable laser photometer DustTrak™ DRX Aerosol Monitor 8533 (TSI, Shoreview, MN, USA), which allowed simultaneous measurements of size-segregated mass fractions corresponding to PM_1_, PM_2.5_, PM_4_, PM_10_ and total PM size fractions. Measurements were carried out five times for 15 min with a sampling interval of 1 s (the number of samples for each measurement was *n* = 900). Dust concentration data were then used to calculate 8-h equivalent time-weighted averages, but as the measurements only lasted for short periods, the calculated values were treated as estimates.

### 2.2. Plant Biomass

Three types of biomass were tested: willow wood chips (taken from the transport/storage tunnel for willow biomass chips), forest wood chips (forest wood biomass tunnel, biomass unloading station and quality control laboratory) and sunflower pellets (sunflower pellet warehouse). Composite samples of willow wood chips and forest wood chips were prepared by mixing 3–6 primary samples (1 kg) collected from evenly distributed places of a storage area from depths 30–150 cm of each prism. In case of sunflower pellets, four composite samples were obtained from the automatic feeder that collects and homogenizes primary samples of biomass from a conveyor belt at specified time intervals before it entered the warehouse. The primary samples of each biomass type were combined and homogenized to obtain collective samples with a volume of 1–2 kg. Collective samples were divided into three laboratory samples of 10 g, from which three analytical samples (1 g) were taken for the tests.

### 2.3. Tested FFRs

Standard reusable FFRs were used in the analysis. These were approved for workplace use in the European Union (EU) in accordance with the provisions of Directive 89/686/EEC, which evaluates the compliance of personal protective equipment (PPE) with basic safety and ergonomic requirements [18]. The filtration efficiency of the FFRs was 99% at a flow rate of 95 L/min against an aerosol of solid particles of sodium chloride and liquid droplets of paraffin oil. FFRs were rated as protective class 3 (FFP3) and were reusable (R) in accordance with the requirements of the European Standard EN 149 [19].

For analysis (microbiological contamination, microscopic and secondary metabolite assessment), all nonwoven materials forming the face piece and the particle filter were used. The tests were conducted for two FFRs used during biomass processing by workers of the combined heat and power plant for three days during 8-h work shifts.

### 2.4. Microbiological Contamination Analysis

Microorganism concentrations were analysed for the air, plant biomass and the reusable FFRs. Microbiological contamination of the air was measured using an MAS-100 Eco Air Sampler (Merck, ‎Darmstadt‎, Germany) according to the EN 13098 standard [20]. Air samples of 50 L and 100 L were collected on malt extract agar (MEA) medium (Merck, Darmstadt‎, Germany) with chloramphenicol (0.1%) to determine the total concentration of fungi (including hydrophilic and xerophilic moulds), and on tryptic soy agar (TSA) medium (Merck, Darmstadt‎, Germany) with nystatin (0.2%) to determine the total concentration of bacteria. Samples of air were collected in four locations at a height of 1.5 m during routine working activities (Table 1). Atmospheric air samples (external background) were collected at a distance of 5 km from each site.

FFR samples, with a surface area of 4 cm^2^, and each of the above described plant biomass samples (six samples weighing 1 g each) were analysed. For this purpose, samples were collected in sterile bins, mixed with 25 mL saline solution (0.85% NaCl) and shaken for 10 min to wash out the microorganisms from the tested materials. The saline solutions containing the microorganisms were then serial diluted (from 10^0^ to 10^6^) and plated on MEA medium with 0.1% chloramphenicol (fungi) and TSA medium with 0.2% nystatin (bacteria).

All samples (air, biomass, FFRs) were incubated at 30 ± 2 °C for 48 h (bacteria) or at 25 ± 2 °C for 5–7 days (fungi). After incubation, the colonies were counted, and the results were expressed in CFU/m^3^ air, CFU/g biomass and CFU/cm^2^ FFRs. The final result was calculated as the arithmetic mean of three independent repetitions.

### 2.5. Identification of Microorganisms

#### 2.5.1. Culture-Dependent Method

All bacteria and yeasts isolated from the samples (air, biomass, FFRs) were transferred onto individual culture plates. Following this, they were macroscopically and microscopically characterized using Gram-staining, catalase test and oxidase test (Microbiologie Bactident Oxidase, Merck, Darmstadt‎, Germany). Next, isolates with the same morphology and biochemical features were grouped into strains and identified using API tests (bioMérieux, Marcy-l’Étoile, France): API 50 CH, API STAPH and API 20 NE (for bacteria) and API C AUX (for yeasts). Isolated filamentous fungi were cultured on Czapek yeast extract agar (CYA, Difco, Franklin Lakes, NJ, USA) and yeast extract with supplements (YES) media and visually identified, macroscopically and microscopically, using taxonomic keys [21,22,23,24].

#### 2.5.2. Next-Generation Sequencing on the Illumina Platform

High-throughput sequencing analysis was performed using the Illumina MiSeq platform to assess microbial diversity on the reusable FFRs. This method was selected as scanning microscopy analysis revealed numerous microorganism cells on the filter materials of the FFRs, while low microbial contamination was detected in the culture method. Thus, we suspected the presence of unculturable microorganisms in these samples.

##### DNA Extraction

DNA was extracted directly from 2 cm^2^ fragments of reusable FFRs. First, small fragments were cut and ground under liquid nitrogen. The FastDNA^®^ Spin Kit (No. 116560200, MP Biomedicals, Santa Ana, CA, USA) was employed for DNA extraction following the manufacturer’s protocol. Quantification of DNA was conducted using Qubit 2.0 Fluorometer (Invitrogen/Life Technologies, Carlsbad, CA, USA).

##### PCR Amplification and Sequencing

Bacterial 16S rRNA gene fragment were amplified using universal primer set 341F and 785R. The amplification of the fungal ITS1 region for high-throughput sequencing analysis was performed with primers given in Table 2.

PCR reactions (both for amplification of 16S rRNA gene fragments and ITS1 regions) were prepared in 50 μL volume containing 5 μL of DNA as template, 25 μL NEBNext^®^ Hot Start High-Fidelity 2× PCR Master Mix (New England BioLabs, Ipswich, MA, USA) and 10 pmol of 341F and 785R (16S rRNA gene) or IT1F12 and ITS2 (ITS1 region) primers. Amplification of bacterial and fungal fragments was performed under the same conditions: an initial denaturation at 98 °C for 30 s was followed by 15 cycles of denaturation at 98 °C for 10 s, annealing at 52 °C for 75 s, extension at 65 °C for 75 s, and a final extension at 65 °C for 5 min. DNA libraries were constructed using Nextera Index kit following the manufacturer’s library preparation protocol for short amplicons (2 × 250 bp). Paired-end (PE, 2 × 250 nt) sequencing was performed on an Illumina MiSeq (MiSeq Reagent kit v2 (Illumina Inc., SanDiego, CA, USA)) sequencer following manufacturer’s run protocols (Illumina Inc., SanDiego, CA, USA) at Genomed, Warsaw, Poland.

##### Sequencing Data Analysis

QIIME (Quantitative Insights Into Microbial Ecology) was used to determine the microbial communities on FFR samples [28]. The raw reads were demultiplexed and quality-filtered on MiSeq using the MiSeq Reporter (MSR) v2.4 (BaseSpace, Illumina Inc., SanDiego, CA, USA). Sequences were clustered at 97% identity with uclust [29] and operational taxonomic units (OTUs) were assigned to taxa using Greengenes database v13_5 for bacteria and UNITE database for fungi [30,31].

### 2.6. Scanning Electron Microscopy (SEM) Method

FFR samples were deposited onto aluminium specimen mounts with carbon tape. The samples were coated with a gold layer using a JFC-1200 fine coater (JEOL Ltd., Tokyo, Japan) and evaporated in an argon atmosphere (argon pressure-8Pa) for 30 s at an amperage of 15 mA. Subsequently, scanning electron microscopy images were acquired on a Nova Nanos 230 (FEI, Hillsboro, OR, USA) with field emission gun (FEG)-type field emission. The test was performed under high vacuum using an accelerating voltage of 5 kV. Registration images of the surface of the tested filtering materials were captured at magnifications of 1500×–6000×.

### 2.7. Analysis of Secondary Metabolites

Secondary metabolite profiles were determined for plant biomass samples and the used FFRs. For the liquid chromatography/tandem mass spectrometry (LC-MS/MS) analysis, 1 g of biomass of each type, and FFR samples (4 cm^2^ surfaces), were suspended in 5 mL of the extraction solvent (acetonitrile/water/acetic acid 79:20:1, v/v/v). Samples were extracted for 90 min and diluted with the same volume of solvent prior to injection [32]. Centrifugation was not necessary as gravity produced sufficient sedimentation. The samples were analysed quantitatively using LC-MS/MS, as described by Sulyok et al. with further modification [33]. The liquid chromatography and mass spectrometry methods and parameters are described elsewhere [34]. Briefly, LC-MS/MS screening of target microbial metabolites was performed with a QTrap 5500 LC-MS/MS System (Applied Biosystems, Foster City, CA, USA) equipped with a TurboIonSpray electrospray ionization (ESI) source and a 1290 Series HPLC System (Agilent, Waldbronn, Germany). Chromatographic separation was performed at 25 °C on a Gemini^®^ C18-column, 150 × 4.6 mm i.d., 5 mm particle size, equipped with a C18 4 × 3 mm i.d. security guard cartridge (Phenomenex, Torrance, CA, USA). ESI-MS/MS was performed in the time-scheduled multiple reaction monitoring (MRM) mode, both in positive and negative polarities, in two separate chromatographic runs per sample, by scanning two fragmentation reactions per analyte. The MRM detection window of each analyte was set to its expected retention times of ±27 s and ±48 s in the positive and negative modes, respectively. Positive analyte identification was confirmed by the acquisition of two MRMs per analyte, with the exception of moniliformin, which exhibited only one fragment ion. This yielded 4.0 identification points according to the European Union Commission decision 2002/657/EC [35]. The LC retention time and the intensity ratio of the two MRM transitions agreed with the related values of an authentic standard within 0.1 min and 30% rel.

### 2.8. Statistical Analysis

Statistical analyses were performed using STATISTICA 12 software (Statsoft, Tulsa, OK, USA). Microorganisms concentration in the air, biomass, FFRs and fraction of dust concentration in particular workplaces were evaluated using one-way analysis of variance (ANOVA) at the 0.05 significance level. When statistical differences were detected (*p* < 0.05), means were compared by the post hoc Tukey’s test at the 0.05 significance level.

Linear regression analysis was used to determine the correlation between microbiological contamination and ambient temperature and relative humidity on of the air in tested workplaces. Linear regression analysis was also used to determine the correlation between overall concentration of microorganisms in the air and their concentration in biomass and FFRs. The significance tests were performed at the 0.05 significance level and Pearson correlation coefficients (r) were calculated. The strength of correlation was interpreted based on the classification described in [36].

## 3. Results and Discussion

The laboratory room of the thermal power station had a temperature of 21 °C and relative humidity of 46%. The spaces dedicated to storage, transport and unloading of biomass had lower temperatures (8–13 °C) and higher levels of humidity (66%–68%). Dust fraction PM_1_, PM_2.5_, PM_4_, and PM_10_ concentrations were significantly different for each tested workplace (*p* < 0.05). Total dust concentration was significantly lowest in tunnel of willow wood chips conveyor and laboratory while the highest was noted in biomass unloading (*p* < 0.05; Table 3). Agitation of the biomass during unloading caused an approximately 3 to 11-fold increase in dust concentration in relation to the locations where biomass was stored during the test (willow and forest wood chips conveyor tunnels).

A limited number of recent studies provide information on dust concentrations measured within facilities associated with biomass processing power stations. The dust concentration values reported in our study were similar to those measured by Rohr et al. [37] during normal operation of two CHP stations, and approximately one order of magnitude lower than those described by Laitinen et al. [38] for stationary site and truck driver’s breathing zones during unloading of wood chips, stumps, peat and solid recovered fuel. Jumpponen et al. reported much higher concentrations of dust (reaching 175 ± 106 mg/m^3^) inside and outside biomass-fired power plant boilers during post-combustion ash removal and other maintenance tasks [39]. These differences could result from the type of performed operations (shredding, transportation, unloading); the temperature and humidity conditions; the characteristics of the area were the tests were performed (open, semi-open or closed space) or the origins of airborne particles (pre-combustion and post-combustion sources).

In all tested areas of the CHP station, the dominant PM fraction had an aerodynamic diameter below 1 μm, and it accounted for 41%–83% of total measured dust. The smallest portion of the total PM constituted particles with diameters between 1 and 4 μm (0%–18%). Dust particles of aerodynamic diameter larger than 10 μm accounted for 7%–25% of total PM, depending on the area. Inhalable dust detected in the CHP can be deposited in lower as well as upper parts of the respiratory system. Smaller particles with diameters above 0.5 μm are deposited in bronchi, bronchioles and alveoli, while large proportion of particles between 5 and 10 μm and most of those larger than 10 μm are trapped in nasopharyngeal region [40].

Depending on the place of deposition, the retention time in the respiratory tract can vary from few hours (for upper respiratory tract) to several hundred days (for lower respiratory tract) [40,41]. This, and the type of the inhaled dust, affects the way in which dust particles interact with human cells. Unfortunately, the information regarding overall exposures of biomass-based CHP workers and epidemiologic studies is limited, which makes it difficult to determine the direct effect of biomass dust on the potential adverse health effects. However, in the case of dust hazards in CHPs, wood dusts have been identified as one of the major causes for concern [37]. In numerous studies, wood dust has been recognized as a respiratory irritant, sensitizer, and toxicant causing pulmonary function impairment [10,42,43], it has been also considered carcinogenic to humans [44,45,46,47,48]. Mandatory or recommended occupational exposure limits (OELs) have been established in a number of countries, based on either total inhalable dust or respirable wood dust of a specific type (e.g., hardwood, softwood, impregnated/non-impregnated wood dust, grain dust) [49,50,51,52]. Threshold limit values (TLVs) for biomass-relevant substances vary from 0.05 mg/m^3^ (Swedish OEL for impregnated wood dust) to 6 mg/m^3^ (Russian OEL for wood dust) [37]. Although the dust concentration values reported in our study (Table 3) are in all cases lower than 1 mg/m^3^, which is lower than the majority of established TLVs, they exceeded the suggested limit values specified in air quality guidelines of World Health Organization (WHO) for airborne particulate matter that are equal to 0.025 mg/m^3^ for PM_2.5_ and 0.050 mg/m^3^ for PM_10_, respectively [53].

The highest concentration of microorganisms was found in plant biomass processed by the CHP, lower *p*-values were observed in the air at all workplaces and the reusable FFRs (*p* < 0.05). Regression analysis revealed high correlations between bacteria concentrations in the air and ambient temperature in corresponding areas (r = −0.897). Likewise, a significant correlation was found between bacteria concentrations and relative humidity (r = 0.824). Only a moderate correlation was observed for fungi concentration in the air and temperature and relative humidity conditions (r = −0.492 and r = 0.560, respectively).

Bacterial concentrations in the biomass ranged from 1.7 × 10^6^–3.5 × 10^7^ CFU/g depending on type, while for the fungal species the concentration was from 3.3 × 10^4^ CFU/g in sunflower pellets to 3.9 × 10^5^ CFU/g in forest wood chips (Table 4).

The concentration of bacteria in the air of the thermal power station was from 5.9 × 10^2^ CFU/m^3^, to 4.6 × 10^3^ CFU/m^3^. The same relationship was found for fungi, the concentrations of which ranged from 6.3 × 10^2^ to 3.8 × 10^4^ CFU/m^3^. Madsen showed a higher concentration of microorganisms in the air of five Danish plant biomass processing biofuel plants (average concentration of fungi was 4.7 × 10^5^ CFU/m^3^, and that of bacteria including, actinomycetes, was 1.5 × 10^6^ CFU/m^3^) [14].

The results obtained are in agreement with the studies of Ławniczek-Wałczyk et al. performed in a power plant processing forest and agricultural biomass [9]. The authors found that the bacterial aerosol ranged from 5.1 × 10^2^ to 3.3 × 10^4^ CFU/m^3^, and fungi between 2.2 × 10^2^ and 2.0 × 10^4^ CFU/m^3^.

The concentration of microorganisms found in the air in this study was similar to the results of other work environments in which plant material is present, including herb, flax, flour and , barley processing plants, and those that process and store grain [54].

The concentration of microorganisms on the FFRs was 1.6 × 10^2^ CFU/cm^2^ (bacteria) and 2.5 × 10^1^ CFU/cm^2^ (fungi). Wang et al. argue that the polymers most commonly used for manufacturing FFRs do not offer any advantages for microbial growth [55]. However, during use the accumulating moisture together with human secretions and dust modify the conditions of the FFRs favouring the growth of microorganisms [55,56]. Although information on microbial growth on FFRs under controlled model conditions can be found in the literature [17,55], there are no data on microorganisms colonising FFRs that are used multiple times in the work environment. We also checked if there was an association between microorganism concentration in the air and biomass or FFRs in the same workplaces. The results indicate a very high correlation (r = 0.995 and r = 0.899) between total concentration of microorganisms in the air and in the stored biomass in tunnel of willow wood chip conveyor and tunnel of forest wood chips conveyor. Similarly, a very high correlation was found (r = 0.997) between the concentration of total microorganisms in the air and FFRs in the laboratory where they were in use. Bacteria dominated amongst the microorganisms isolated from all types of biomass and the FFRs, whereas fungi predominated in the air of willow wood chips conveyor tunnel and in the biomass unloading zones.

The culture method detected 8 mesophilic genera of bacteria and 7 genera of fungi in the air, 10 genera each of bacteria and fungi in the biomass, and 2 genera of bacteria and 5 of fungi on the FFRs.

In the air of the thermal power station, the highest percentages of bacterial content corresponded to: *Moraxella* sp. (43%), *Bacillus megaterium* (27%), *Cellulomonas* sp. (23%) and *Penicillium chrysogenum* (86%). On the other hand, the biomass samples had *Staphylococcus sciuri* (79%) and *Aeromonas hydrophila* (18%), *Rhodotorula mucilaginosa* (34%), *Rhizopus nigricans* (12) and *Paecilomyces variotii* (11%) (Table 5). The FFRs mainly contained the bacteria *Bacillus subtilis* (84%) and *Staphylococcus hominis* (18%), as well as the moulds *Mucor racemosus* (54%), *Rhizopus nigricans* (21%) and *Cladosporium cladosporioides* (18%) (Table 5).

Many of the species (29% of bacteria and 27% of fungi) isolated from biomass were also present in the air, which suggests that plant biomass is one of the main sources of microorganisms in the bioaerosol in the CHP. Sporulating *B. subtilis* was the only bacteria found in all “elements” (biomass, air and FFRs) tested. Amongst the isolated microorganisms were species potentially harmful to human health, such as *Vibrio* sp., that belongs to group 2 health hazards according to the classification outlined in Directive 2000/54/EC [57].

**Figure 1:**
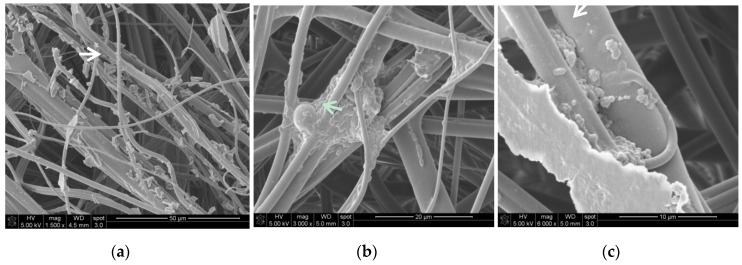
Biofilm on reusable FFR: (**a**) FFR fibres with visible dust particles; (**b**) bacterial and fungal biofilm with extracellular polymeric substances (EPS); (**c**) bacterial biofilm coexisting with organic dust particles (SEM images 1500–6000×).

The possibility of microbial colonization on the reusable FFRs used in the thermal power station was also examined by scanning electron microscopy (Figure 1). SEM analysis showed that microorganisms grew in the form of a biofilm on these FFRs. Bacterial cells, extracellular polymeric substances (EPS) and dust particles from biomass were observed on the surfaces. The presence of fungal structures, probably of moulds of the *Rhizopus* or *Mucor* genera, were detected by SEM and culture methods (Figure 1b, Table 5).

The evaluation of microorganism species present on reusable FFRs was performed for the first time using next generation sequencing technique on the Illumina MiSeq platform. High-throughput sequencing has been previously employed to characterize the bacterial and fungal communities in different environments such as soil, compost, oilfield, animal and human gut, marine and freshwater, and cultural heritage materials [58]. DNA sequencing experiments identified 46 genera of bacteria that belong to 8 phyla and only 5 genera of fungi from 2 phyla on the FFRs (Table 6). The FFR sample was dominated by *Firmicutes* and *Proteobacteria* phyla that constituted 64.2% and 23.8% of operational taxonomic units (OTU), respectively. Amongst *Firmicutes*, bacteria from *Bacillus* (43.4%), *Paenibacillus* (14.3%) and *Weissella* (3.0%) genera were identified. Moreover, DNA sequences characteristic of *Staphylococcus* (0.74%), *Lactobacillus* (0.72%) and *Geobacillus* (0.6%) that belong to this phylum were also detected (Table 6). Amongst *Proteobacteria*, the most abundant class was *Betaprotobacteria* contributing *Ralstonia* (17.0%), *Burkolderia* (1.2%) and *Thauera* (0.54%). The third most predominant phylum in the FFR samples was *Actinobacteria* that constituted 5.5% of the total bacterial OTU with a prevalence of *Propionobacterium* (2.8%), *Arthrobacter* (1.1%), *Micrococcus* (0.4%) *Corynebacterium* (0.4%) and *Streptomyces* (0.2%) genera. The bacterial population was also represented by minor phyla (*Acidobacteria*, *Bacteroidetes*, *Cyanobacteria*, *Nitrospirae* and *Planctomycetes*) with a less than 1% contribution (Table 5).

Fungal diversity was significantly lower than that of bacteria colonising FFRs. The sample content was dominated by mould species from genera: *Aspergillus* (51.1%) and *Penicillium* (2.3%) from the *Eurotiomycetes* class. Fungi *Saccharomycetes* constituted only 1.2% of total OTU and were represented by yeast from *Candida* and *Nakazawaea* genera (Table 6). It is worth noting that 19.36% of the OTU was composed of unclassified fungi, whereas unclassified bacteria constituted only 6.9%.

Among the microorganisms detected in FFRs, saprophytic species typical of the skin and mucous membranes of human origin were identified, i.e., *Acinetobacter*, *Brevibacterium*, *Corynebacterium*, *Dermabacter*, *Micrococcus*, *Propionibacterium*, *Rhodococcus*, *Staphylococcus*, *Streptococcus* [59].

However, the presence of *Candida tropicalis*, *Corynebacterium* sp., *Escherichia coli* and *Prevotella* sp. in FFRs, is of particular importance, as those species belong to group 2 health hazards according to the Directive 2000/54/EC [57]. It should be emphasized that the Illumina technique enabled the detection of moulds from the *Aspergillus* and *Penicillium* genera, which include many allergenic and toxinogenic species.

Illumina sequencing allowed us to detect a larger variety of bacteria on the FFRs compared to culture methods. In addition, this method detected more harmful biological agents than culture alone.

Depending on biomass type, between 22 and 27 secondary metabolites were detected. In all types of biomass 3-nitropropionic acid, alternariolmethylether, ascochlorin, citreorosein, emodin, integracin B, skyrin and quinocitrinine A were found at concentrations ranging from 0.08 to 180.9 mg/kg (Table 7). It is noteworthy that high concentrations of fumigaclavine and fumiquinazoline compounds were present only in forest wood chips and sunflower pellets. On the other hand, infectopyron, orsellinic acid, sterigmatocystin, tentoxin, tenuazonic acid were present in willow wood chips, but were not detected in other types of biomass.

Five secondary metabolites were uncovered on the reusable FFR samples. They were: altersetin, asperglaucide, citreorosein, emodin and fallacinol, at concentrations between 1.67 and 399.9 μg/kg (Table 7). With the exception of fallacinol, these compounds also occurred in all plant biomass samples. 

We found metabolite characteristics associated to the following genera in biomass and on reusable FFRs: *Alternaria* (alternariol, alternariolmethylether, altersetin, infectopyrone, macrosporin, tentoxin, tenuazonic acid), *Aspergillus* (3-nitropiopropionic acid, averantin, averufin, fumigaclavine C, fumigaclavine, fumiquinazolin A and D; fumiquinazolin F, fumitremorgin C, methylsulochrin, sydonic acid, sterigmatocystin, versicolorin C, norsolorinic acid, pseurotin A, pyripyropene A), *Cladosporium* (cladosporin) and *Penicillium* (cyclopenol, viridicatum toxin, andrastin A). In addition, nonspecific metabolites, the occurrence of which cannot be linked to specific moulds, such asperglaucide, brevianamid F, citreorosein, emodin, enniatin B, enniatin B1, neoechinulin A, were identified. Secondary mould metabolites may be due to contamination from biomass that, with air movement, gets deposited on the FFRs. It is worth emphasising that secondary metabolites are stable in the environment and can be detected even after the mould dies. This may be the reason for detecting metabolites of *Alternaria*, despite us not isolating any moulds of this genus by culture methods. In addition, we also detected metabolites that are characteristic of *Aspergillus* and *Penicillium* moulds, within the pool of unculturable microorganisms on the FFRs. The results show that the detection of moulds must be performed using both culture and metagenomic methods.

Importantly, we detected mycotoxins, which may have a negative impact on human health: fumigaclavines may affect the central and peripheral nervous systems; quinocitrinines indicate antibiotic antitumor activity; sterigmatocistin has carcinogenic potency; and 3-Nitropropionic acid causes biochemical and morphological alterations in human and animal brains [60,61,62].

## 4. Conclusions

Biomass processed in a thermal power station is a source of organic dust that contains microorganisms and mycotoxins floating in the air of the plant. They pose a health threat to power plant staff. The majority of dust and microbiological contamination of air occurs during the unloading of biomass. Air contamination also occurs in biomass storage areas and in the laboratory, albeit at lower levels. We detected potential pathogens, such as *Candida tropicalis*, *Escherichia coli*, and *Prevotella* sp. on the reusable FFRs used by power plant staff. In addition, fungi from the *Aspergillus* and *Penicillium* genera were found. We also detected secondary metabolites, including fumigaclavines, quinocitrinines, sterigmatocistin, 3-nitropropionic acid, which may be toxic to humans, in the biomass and on the reusable FFRs. 

The ability of microorganisms to grow on the FFRs was confirmed using multiple methods (culture, SEM and Illumina MiSeq sequencing). Furthermore, Illumina sequencing enabled us to detect a larger variety of microorganisms than would have been possible if culture methods were used on their own. Thus, employing the above methods in conjunction with secondary metabolite analysis would be beneficial for the identification of microorganism, especially fungi, in such workplaces/samples.

Due to the high concentration of microorganisms and the presence of potential pathogens and mycotoxins, the level of microbiological contamination in workplaces at power plants processing biomass should be monitored. Based on our research, we believe that innovative solutions, which prevent microorganism colonisation of FFRs that can be used multiple times, must be developed. Until then, special maintenance procedures for reusable FFRs should be proposed. In particular, they should include the information on the maximum number of uses, storage conditions (type of packaging, temperature and humidity of storage) as well as cleaning and/or disinfection method depending on the workplace characteristics and the type of biological hazard. In case of especially harmful biological agents, it can be also beneficial for the workers’ health to consider the use of non-reusable respiratory protective equipment.

## Figures and Tables

**Table 1:** Characteristics of selected workplaces at the thermal power station processing biomass.

Workplace	Description of Performed Activities	Premise Size (Length/Width/Height) (m)	Air Flow Rate M ± SD (m/s)
Tunnel of willow wood chips conveyor	Biomass is transported to a warehouse, unloaded and stored. During storage, workers measure the humidity and temperature of the prism. The biomass is then appropriately processed for energy generation.	9.0/4.7/3.9	0.19 ± 0.01
Tunnel of forest wood chips conveyor		80.0/3.5/4.1	0.13 ± 0.02
Biomass unloading station	Following the delivery of the biomass to the plant, workers unload it. This disperses organic dust into the air.	6.3/9.4/9.5 *	0.13 ± 0.07
Laboratory	Biomass undergoes initial processing (mincing, homogenising, drying) and is then subjected to laboratory analyses.	5.1/5.5/2.9	0.03 ± 0.03

* semi-open area; M: mean; SD: standard deviation.

**Table 2:** Sequences of primers used in this study.

Amplified Sequence	Primer Name	Primer Sequence (5’ > 3’)	Reference
16S rRNA	341F 785R	CCTACGGGNGGCWGCAG GACTACHVGGGTATCTAATCC	Klindworth et al. [25] Klindworth et al. [25]
ITS1 region	ITS1F12 ITS2	GAACCWGCGGARGGATCA GCTGCGTTCTTCATCGATGC	Schmidt et al. [26] White et al. [27]

**Table 3:** Microclimate characteristics and dust concentration at workplaces in the power plant.

Workplace	Air Temperature (°C)	Relative Humidity M ± SD (%)	Concentration of Dust Fraction (mg/m^3^) M ± SD				
			PM_1_	PM_2.5_	PM_4_	PM_10_	PM_total_
Tunnel of willow wood chips conveyor	9.2 ± 0.6	68.3 ± 3.5	0.070 ± 0.015 ^**a**^	0.071 ± 0.015 ^**a**^	0.073 ± 0.015 ^**a**^	0.078 ± 0.018 ^**a**^	0.085 ± 0.026 ^**a**^
Tunnel of forest wood chips conveyor	12.8 ± 0.6	66.1 ± 4.8	0.148 ± 0.049 ^**b**^	0.158 ± 0.052 ^**b**^	0.190 ± 0.053 ^**b**^	0.260 ± 0.060 ^**b**^	0.296 ± 0.071 ^**b**^
Biomass unloading	8.5 ± 0.3	68.4 ± 3.1	0.395 ± 0.448 ^**c**^	0.460 ± 0.564 ^**c**^	0.571 ± 0.711 ^**c**^	0.815 ± 1.100 ^**c**^	0.967 ± 1.280 ^**c**^
Laboratory	20.6 ± 2.5	46.2 ± 6.3	0.030 ± 0.022 ^**d**^	0.030 ± 0.022 ^**d**^	0.030 ± 0.22 ^**d**^	0.032 ± 0.024 ^**d**^	0.043 ± 0.039 ^**a**^

PM_1_; PM_2.5_; PM_4_; PM_10_ respectively: granulometric fraction with a diameter less than 1; 2.5; 4; 10 μm; M: mean; PM: particulate matter; SD: standard deviation; ^**a**, **b**, **c**, **d**^: means (among dust fraction) that do not share a letter are significantly different (one-way ANOVA, *p* < 0.05; Tukey’s test, *p* < 0.05).

**Table 4:** Concentration of microorganisms isolated from the air, biomass samples and filtering facepiece respirators (FFRs).

Area/Sample Tested	Concentration of Microorganisms	
	Bacteria	Fungi
Air (CFU/m^3^)		
Tunnel of willow wood chips conveyor	M: 2.67 × 10^3 **c**^ Min: 2.39 × 10^3^ Max: 2.99 × 10^3^ SD: 3.02 × 10^2^	M: 1.67 × 10^3 **b,c**^ Min: 1.60 × 10^3^ Max: 1.72 × 10^3^ SD: 6.43 × 10^1^
Tunnel of forest wood chips conveyor	M: 2.27 × 10^3 **c**^ Min: 2.18 × 10^3^ Max: 2.37 × 10^3^ SD: 9.61 × 10^1^	M: 3.25 × 10^4 **b,c**^ Min: 2.63 × 10^4^ Max: 4.20 × 10^4^ SD: 8.33 × 10^3^
Biomass unloading	M: 4.57 × 10^3 **c**^ Min: 4.02 × 10^3^ Max: 5.20 × 10^3^ SD: 5.94 × 10^2^	M: 3.79 × 10^4 **b,c**^ Min: 2.63 × 10^4^ Max: 5.10 × 10^4^ SD: 1.24 × 10^4^
Laboratory	M: 5.93 × 10^2 **c**^ Min: 8.00 × 10^1^ Max: 8.80 × 10^2^ SD: 4.45 × 10^2^	M: 6.30 × 10^2 **b,c**^ Min: 4.40 × 10^2^ Max: 7.70 × 10^2^ SD: 1.71 × 10^2^
External background	M: 2.03 × 10^2 **c**^ Min: 1.20 × 10^2^ Max: 3.30 × 10^2^ SD: 1.12 × 10^2^	M: 2.03 × 10^2 **b,c**^ Min: 1.80 × 10^2^ Max: 2.30 × 10^2^ SD: 2.52 × 10^1^
Biomass (CFU/g)		
Willow wood chips	M: 3.51 × 10^7 **a**^ Min: 2.73 × 10^7^ Max: 4.40 × 10^7^ SD: 7.24 × 10^6^	M: 2.91 × 10^5 **a,b**^ Min: 2.00 × 10^5^ Max: 4.76 × 10^5^ SD: 1.05 × 10^5^
Forest wood chips	M: 2.28 × 10^7 **b**^ Min: 1.16 × 10^7^ Max: 3.61 × 10^7^ SD: 9.78 × 10^6^	M: 3.86 × 10^5 **a,b**^ Min: 1.64 × 10^5^ Max: 8.20 × 10^5^ SD: 3.16 × 10^5^
Sunflower pellets	M: 1.68 × 10^6 **c**^ Min: 1.00 × 10^6^ Max: 3.29 × 10^6^ SD: 8.69 × 10^5^	M: 3.29 × 10^4 **c**^ Min: 1.15 × 10^4^ Max: 7.80 × 10^4^ SD: 2.62 × 10^4^
FFR (CFU/cm^2^)		
FFR	M: 1.62 × 10^2 **c**^ Min: 1.83 × 10^1^ Max: 3.84 × 10^2^ SD: 1.24 × 10^2^	M: 2.54 × 10^1 **c**^ Min: 8.50 × 10^0^ Max: 5.75 × 10^1^ SD: 1.71 × 10^1^

M: mean; Max: maximum value; Min: minimum value; SD: standard deviation; ^**a**, **b**, **c**^: means (among groups of microorganisms) that do not share a letter are significantly different (one-way ANOVA, *p* < 0.05; Tukey’s test, *p* < 0.05). FFR: filtering facepiece respirator.

**Table 5:** Microorganisms isolated from the air, biomass and FFR in cultivation method.

Domain	Species	Percentage (%)		
		Air	Biomass	FFR
**Bacteria**	*Aeromonas hydrophila*	-	17.83	-
	*Bacillus cereus*	1.68	-	-
	*Bacillus megaterium*	26.59	-	0.18
	*Bacillus pumilus*	-	0.01	-
	*Bacillus subtilis*	2.24	0.11	84.48
	*Brevundimonas vesicularis*	2.10	0.52	-
	*Cellulomonas* sp.	22.88	-	-
	*Micrococcus* sp.	0.84	-	-
	*Moraxella* sp.	42.90	0.14	-
	*Pseudomonas fluorescens*	-	0.42	-
	*Pseudomonas luteola*	0.14	0.15	-
	*Sphingomonas multivunum*	-	0.43	-
	*Sphingomonas paucimobilis*	-	0.14	-
	*Staphylococcus hominis*	0.42	-	18.33
	*Staphylococcus sciuri*	-	78.90	-
	*Streptomyces* sp.	0.21	0.20	-
	*Vibrio metschnikovii*	-	1.1	-
**Fungi**	*Acremonium* sp.	-	0.11	-
	*Aureobasidium pullulans*	-	0.09	-
	*Candida famata*	2.83	-	-
	*Candida lusitaniae*	0.38	-	-
	*Chrysonilia sitophila*	0.53	-	3.72
	*Cladosporium cladosporioides*	1.06	7.59	18.33
	*Mucor hiemalis*	2.93	-	-
	*Mucor racemosus*	-	0.26	54.85
	*Paecilomyces variotii*	-	10.56	-
	*Penicillium chrysogenum*	86.08	6.54	-
	*Penicillium commune*	4.22	-	-
	*Rhizopus nigricans*	0.86	12.25	21.91
	*Rhodotorula mucilaginosa*	-	33.67	-
	*Trichoderma viride*	-	8.46	-
	*Verticillium tenerum*	1.10	20.45	1.20

- : not detected.

**Table 6:** The assessment of microbial species composition in FFR samples.

Domain	Phylum	Genus	Abundance of Bacterial/Fungal Genera in Samples (%)
**Bacteria**	*Acidobacteria*	*Acidobacterium*	0.06
		Other	0.05
	*Actinobacteria*	*Arthrobacter*	1.05
		*Corynebacterium*	0.38
		*Micrococcus*	0.41
		*Nonomuraea*	0.10
		*Propionibacterium*	2.83
		*Rubrobacter*	0.20
		*Streptomyces*	0.14
		Other	0.38
	*Bacteroidetes*	*Chitinophaga*	0.18
		*Chryseobacterium*	0.25
		*Prevotella*	0.19
		Other	0.14
	*Cyanobacteria*	*Leptolyngbya*	0.53
	*Firmicutes*	*Ammoniphilus*	0.22
		*Bacillus* *	43.35
		*Desulfosporomusa*	0.14
		*Geobacillus*	0.60
		*Laceyella*	0.13
		*Lactobacillus*	0.72
		*Paenibacillus*	14.33
		*Staphylococcus* *	0.74
		*Streptococcus*	0.14
		*Thermoactinomyces*	0.25
		*Thermobacillus*	0.15
		*Weissella*	3.00
		Other	0.42
	*Nitrospirae*	*Nitrospira*	0.07
	*Planctomycetes*	*Singulisphaera*	0.12
	*Proteobacteria*	*Acetobacter*	0.16
		*Achromobacter*	0.11
		*Acinetobacter*	0.22
		*Azospirillum*	0.26
		*Burkholderia*	1.18
		*Chromohalobacter*	0.13
		*Curvibacter*	0.15
		*Delftia*	0.14
		*Dyella*	0.11
		*Escherichia*	0.18
		*Helicobacter*	0.20
		*Lysobacter*	0.15
		*Oscillospira*	0.16
		*Pseudomonas*	0.16
		*Ralstonia*	16.98
		*Sphingomonas*	0.61
		*Thauera*	0.54
		*Thermomonas*	0.15
		*Xanthomonas*	0.18
		Other	0.86
	Unclassified		6.10
**Fungi**	*Ascomycota*	*Aspergillus*	51.06
		*Candida* *	0.70
		*Nakazawaea*	0.45
		*Penicillium* *	2.26
		Other	2.21
	*Basidiomycota*	*Malassezia*	0.19
		Other	23.77
	Unclassified		19.36

* genera detected both with culture and Illumina MiSeq methods.

**Table 7:** Secondary metabolites detected in biomass and FFR.

Secondary Metabolite	Concentration of Secondary Metabolites (μg/kg)			FFR
	Willow Wood Chips	Forest Wood Chips	Sunflower Pellets	
3-Nitropropionic acid	11.6	1.96	2.73	-
Alternariol	22.3	-	66.43	-
Alternariolmethylether	6.09	2.65	50.67	-
Ascochlorin	0.77	4.16	4.00	-
Altersetin	-	-	90.63	3.39
Asperglaucide	22.3	0.229	-	399.9
Andrastin A	-	-	1878.2	-
Averantin	-	1.67	1.24	-
Averufin	0.11	1.28	-	-
Citreorosein	5.70	37.12	83.57	14.44
Cladosporin	3.44	-	-	-
Cyclopenol	8.10	-	-	-
Emodin	4.37	45.59	180.9	15.66
Fallacinol	-	-	-	1.67
Fumigaclavine C	-	60,698	33,479	-
Fumigaclavine A	-	12.55	7.52	-
Fumiquinazolin A	-	1959	-	-
Fumiquinazolin D	-	21,073	11,992	-
Fumiquinazolin F	-	6586	3599	-
Fumitremorgin C	-	3.15	-	-
Infectopyron	35.27	-	-	-
Integracin A	-	1.99	5.97	-
Integracin B	0.08	3.50	42.35	-
Isofusidienol	-	-	30.46	-
Macrosporin	6.85	10.42	-	-
Methylsulochrin	-	59.72	40.75	-
Monocerin	-	1.11	145.59	-
Neoechinulin A	58.92	0.39	-	-
Norsolorinic acid	-	10.82	0.723	-
Orsellinic acid	3795	-	-	-
Pseurotin A	-	16.33	30.02	-
Quinocitrinine A	2.90	0.130	1.80	-
Pyripyropene A	-	3.10	-	-
Skyrin	0.489	30.92	32.81	-
Sydonic acid	-	-	5.94	-
Sterigmatocystin	0.694	1.03	-	-
Tentoxin	47.16	-	-	-
Tenuazonic acid	939.5	-	-	-
Viridicatum toxin	2.09	-	-	-
Versicolorin C	-	60.87	-	-

- : not detected.
